# “Ambulatory Management of Moderate to High Risk COVID-19 Patients: The Coronavirus Related Outpatient Work Navigators (CROWN) Protocol”

**DOI:** 10.1177/1084822320964196

**Published:** 2020-10-15

**Authors:** Gita Lisker, Mangala Narasimhan, Harly Greenberg, Ramona Ramdeo, Thomas McGinn

**Affiliations:** 1 Donald and Barbara Zucker School of Medicine at Hofstra/Northwell, New Hyde Park, NY, USA; 2 Donald and Barbara Zucker School of Medicine at Hofstra/Northwell, Great Neck, NY, USA; 3 Donald and Barbara Zucker School of Medicine at Hofstra/Northwell, Manhasset, NY, USA

**Keywords:** COVID-19, homecare, ambulatory, telehealth, outpatient, protocol

## Abstract

During the height of the novel 2019 coronavirus disease (COVID-19) pandemic in New York City, area hospitals were filled to 150% capacity, and there was a significant fear among the public of going to the hospital. Many hospitalized patients were treated with therapies that could be administered in a home setting under proper monitoring. We designed the CROWN Program, a Home-Care based ambulatory protocol to evaluate, monitor, and treat moderate to high risk COVID-19 patients in their homes, with escalation to hospital care when necessary. Patients were evaluated with telehealth visits with a Pulmonologist, and a Home-Care protocol, including RN visit, pulse-oximetry, and oxygen, lab-work, intravenous fluids, medication if needed patient data, comorbidities, and symptoms were collected. Labs, including COVID-19 PCR, D Dimer, CRP, Ferritin, Procalcitonin, CBC, and metabolic panel were measured, as were homecare, home oxygen, and intravenous fluids orders, radiographic studies and initiation of an anticoagulant. Emergency Department visits and need for hospital admission during the study period were recorded. A total of 182 patients were enrolled between the start date of April 27th and June 1st, and fell into two categories: not-admitted (101) and post-discharge (81). Two patients were referred for hospital admission, seven were treated and released from the ED, and one was referred to home hospice. There were no unexpected admissions or deaths. The CROWN program has demonstrated the feasibility and apparent safety of a specialized, Home-Care based protocol for the ambulatory management of moderate to high risk COVID-19 patients.

## Introduction

During the height of the novel 2019 coronavirus disease (COVID-19) pandemic in New York City (NYC) in March and April 2020, area hospitals were filled to 150% capacity, with makeshift hospital wards and shortages of hospital equipment and staff. Single day totals for NYC reached 6,372 cases and 1,721 hospital admissions on April 6th.^1^ Additionally, there was a significant fear among the public of going to the hospital, both for fear of contracting the virus as well as the fear of being alone without visitation. Between March 11th and May 2nd 2020, the New York City Department of Health and Mental Hygiene recorded 32,107 deaths, 24,172 of which were estimated to be in excess of the expected number for that time period.^2^ Though the majority of the excess deaths were attributed to confirmed or suspected COVID-19 infection, 22% of the deaths were not.^2^ Other factors, such as fear of obtaining health or lifesaving care likely played a role. New York City Emergency Services reported an eight-fold increase in the number of dead-on-arrival (DOA) calls during this time-period.^3^ Many patients admitted to medical floors of the hospital were treated with oxygen via nasal cannula, oral medications and intravenous fluids, all of which could be administered in a home setting under proper monitoring. We designed a homecare based ambulatory protocol to evaluate, monitor, and treat moderate to high risk COVID-19 patients in their homes safely, with escalation to hospital care when necessary.

## Methods

**Figure 1.**
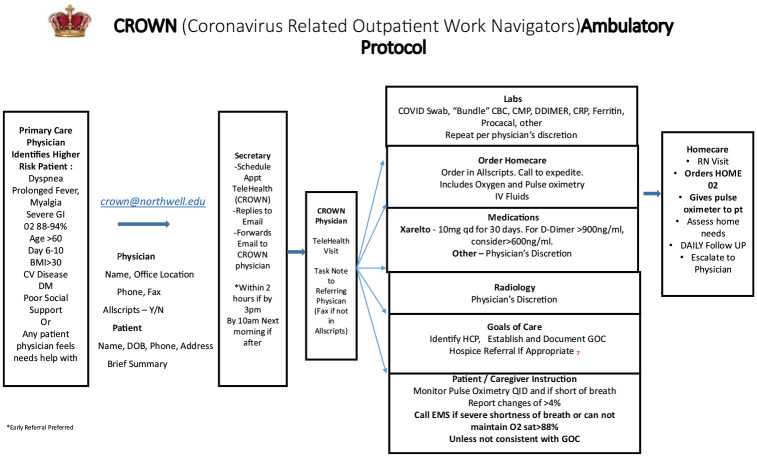
CROWN protocol.

A specialized, multidisciplinary, referral-based protocol for the ambulatory treatment of moderate to high risk COVID-19 patients, entitled the Coronavirus Related Outpatient Care Navigator (CROWN) Program was designed (Figure 1). Participating CROWN providers were pulmonologists from all over metropolitan NY. Our health system is a 12 acute care hospital system with locations in Westchester county, Manhattan, Queens county, Nassau, and Suffolk counties. Our CROWN providers encompassed these regions.

Primary care physicians who identified a COVID-19 patient as moderate to high risk for decompensation or poor outcome could refer the patient to the CROWN program with an email to crown@northwell.edu. Emails were answered by designated office staff, and tele-health appointments with CROWN providers were scheduled for the same day if received by 3 pm and next day if received later, and the service was available 7 days a week. The CROWN providers could also self-refer their own patients to the program.

Moderate to high risk patients were identified as patients with significant comorbidities, advanced age, poor social support, and/or those with moderate to severe symptoms. Patients were scheduled with telehealth visits with a CROWN provider, who then enrolled the patient in the CROWN program and initiated the protocol (Figure 1). Homecare referral included a home visit with a nurse, a pulse-oximeter was provided to the patient, and parameters for ordering home oxygen and intravenous fluid were provided, if needed. Parameters for necessary escalation of care included respiratory distress and/or the inability to maintain an oxygen saturation of >88% despite supplemental oxygen, unless this was not consistent with the patient’s goals of care. Home labs included a COVID-19 polymerase chain reaction (PCR) swab if not already done, a COVID-19 “Bundle” of labs (CBC, Comprehensive Metabolic Panel, D Dimer, CRP, Ferritin, procalcitonin) and other labs felt appropriate by the provider, including a COVID-19 IgG. A Chest Xray was offered as an in-home study, other radiology services were done at one of the facilities, and the patients were identified as CROWN to assure appropriate infection control measures. Medication guidelines included prophylactic dose rivaroxaban for patients with elevated D Dimer levels if no contraindications existed, and it was provided by the hospital pharmacy if it was not covered by the patient’s insurance company. Other medication prescriptions were based on physician discretion.

Health care proxies and goals of care were identified, when appropriate, and referral to palliative care medicine and hospice services were available. Patients and their caregivers were given instructions for monitoring pulse-oximetry and symptoms, as well as when to call the physician or emergency medical services. Follow-up telehealth visits were scheduled based on physician discretion, and the referring providers were kept up to date with notes in the electronic medical record (EMR), secure emails, phone calls or some combination thereof.

The CROWN program went into effect on April 27th, and 182 patients were enrolled between April 27th and June 1st. All patients who were admitted into the CROWN program during that time period were included in this analysis. The Northwell Health Institutional Review Board approved this case series as minimal-risk research using data collected for routine clinical practice and waived the requirement for informed consent. Patient characteristics, CROWN interventions, results and outcomes were collected by retrospective chart review. Data was collected from the enterprise electronic health record (Sunrise Clinical Manager; Allscripts) reporting database. Race and ethnicity data were collected by self-report in prespecified fixed categories.

## Results

Patient referrals fell into two categories: Not admitted and Admitted. About 101 patients were ambulatory patients who were not admitted to a hospital. Eighty-two patients were admitted to a hospital for COVID-19 infection prior to referral, but remained moderate to high risk for a complication or readmission post-discharge.

## Patient Characteristics

Among the 101 patients in the not admitted group, dyspnea was the reason for referral in 67 of them. Other indications for referral included persistent cough or fever, altered mental status, poor oral intake, chest tightness, and severe fatigue. Nine patients had been treated and released from an emergency department (ED) prior to referral. The average age of referred patients was 57 years old, ranging from age 24 to 94. Seventy-four patients were female and 27 were male. Average body mass index (BMI) was 30.2. Forty-seven patients were White, 15 were Black, five were Hispanic, four were Asian, and the others were unknown or declined. Per the EMR records, 31 patients had a history of hypertension, 27 had asthma, 21 had diabetes, 13 had obstructive sleep apnea (OSA), 12 had chronic obstructive pulmonary disease (COPD), and seven had coronary artery disease (CAD) (Table 1). Three patients had a history of oxygen dependence prior to infection and three patients were pregnant. The average time from symptom onset to referral was 26 days, with a range from 1 to 75 days, and a standard deviation of 18 days.

**Table 1.** Patient Characteristics.

	Not admitted (*n* = 101)		Post-discharge (*n* = 81)	
Age	57 (24-94)		62 (28-100)	
Sex	Male	27	Male	40
	Female	74	Female	41
Ethnicity	White	47	White	34
	Black	15	Black	12
	Hispanic	5	Hispanic	12
	Asian	4	Asian	7
	Other/Declined	30	Other/Declined	16
BMI	30.2 (16.4-60)		31 (18-66)	
Comorbidities	Obesity	14	Obesity	15
	Diabetes	21	Diabetes	31
	HTN	31	HTN	43
	COPD	12	COPD	11
	Asthma	27	Asthma	11
	OSA	13	OSA	11
	Cardiac	7	Cardiac	13

Among the 81 post-discharge patients, persistent dyspnea (62) and need for home oxygen (43) were the most common reasons for referral. The average age of the patients was 62, ranging in age from 28 to 100 years old. Forty-one patients were female, 40 were male. Average BMI was 31. Thirty-four patients were White, 12 were Black, 12 Hispanic, 7 Asian, and others unknown or declined. Forty-three patients had a history of hypertension, 31 diabetes, 13 CAD, 11 COPD, 11 asthma, and 11 had OSA (Table 1). Two patients had a history of oxygen dependence prior to admission and 43 of the patients were discharged from the hospital with new supplemental oxygen. A total of seven patients were diagnosed with a thromboembolic event during their admission, six of whom were discharged on full-dose anticoagulation with rivaroxaban. Two patients were on pre-existing full dose apixaban for atrial fibrillation. Fourteen patients were discharged on prophylactic-dose anticoagulation, 12 rivaroxaban and two apixaban. The average time from the positive PCR test to CROWN referral was 34 days, ranging from 10 to 64 days.

## CROWN Interventions and Results

Among the 101 patients in the not admitted group, homecare, including a pulse oximeter, was ordered for 35 patients, oxygen was ordered for 12 patients, labs ordered for 76 patients, radiographic testing was ordered in 14 patients, and intravenous fluids were ordered for five patients (Table 2). Seventy patients had a positive COVID-19 PCR, 23 had a negative COVID-19 PCR, and eight were presumed positive but never tested. Nine of the COVID-19 negative PCR patients subsequently also had negative COVID-19 IgG and were therefore deemed not to be COVID-19 patients. Two patients with a positive COVID-19 PCR were negative for IgG. The D Dimer was normal in 23 patients, greater than 600 ng/mL in 10 patients, with an average of 369 ng/mL and a highest level of 3,430 ng/mL. CRP was normal in 18 patients, with an average of 0.7 mg/dL and a highest level of 5.46 mg/dL. Average ferritin was 211 ng/mL, with a high of 1,806 ng/mL. CTPA was performed on seven patients, all of which were negative for pulmonary embolism, but three of which demonstrated bilateral ground glass opacities. Non-contrast CT chest was ordered on two patients, with bilateral ground glass opacities. Chest X rays were ordered for three patients, one of which was normal, two showed bilateral opacities. Lower extremity Dopplers were negative on both patients ordered. Overall, 11 patients were placed on prophylactic-dose anticoagulation, 10 on rivaroxaban 10 mg and 1 on apixaban 2.5 mg. Ten patients were placed on aspirin. Four patients were already on warfarin for pre-existing conditions. One patient was found to be subtherapeutic on warfarin and was bridged with lovenox. On average, patients had three CROWN tele-visits with the physician during this time period. One patient, a 92 year old woman with advanced dementia, was referred to home hospice and subsequently died.

**Table 2.** Crown Interventions.

	Not admitted* (*N* = 101)	Post discharge** (*n* = 81)
Homecare	35	22
Oxygen	12	2
Labs	76	59
IV fluid	5	1
Hospice	1	0
NOAC prophylaxis	11	7
NOAC therapeutic	0	2
Aspirin	10	0

* In addition to previously ordered by referring physician.

** In addition to those ordered at time of discharge.

Among the 81 patients in the post-discharge group, homecare and oxygen were ordered on 22 and two patients, respectively, if not already ordered at the time of discharge (Table 2). Intravenous fluids were ordered on one patient and labs were ordered on 59 patients. The D Dimer was normal in eight patients, greater than 600 ng/mL in 10 patients, with an average level of 771 ng/mL and a high level of 9,966 ng/mL. Average and highest CRP were 0.99 mg/dL and 10.22, respectively, and average and highest ferritin were 375 and 1,917 ng/mL, respectively. Eight CTPA studies were ordered during this time period, all of which demonstrated bilateral ground glass opacities and one of which demonstrated an acute pulmonary embolism in a patient not being treated with anticoagulation. One lower extremity Doppler was ordered and was negative. Two patients were started on full-dose rivaroxaban—one that was diagnosed with a pulmonary embolism on admission but was discharged only on prophylactic dose anticoagulation, and the one patient with the positive CTPA study. Seven patients were started on prophylactic dose anticoagulation by the CROWN provider. On average, patients had 2.3 tele-visits with a CROWN physician during this time period.

## Outcomes

Among the 101 not admitted patients, two patients were referred for hospital admission by a CROWN physician, one for an INR of 13 and one for severe dyspnea. Four patients were treated and released from the ED without intervention following CROWN referral, and all were related to anxiety based on the ED physician notes. The only death was one patient who was referred to home hospice.

Among the 81 post discharge patients, there were no re-admissions during this time period. Three patients were treated and released from the ED without intervention. There were no deaths.

## Discussion

With the implementation of our CROWN program, we have demonstrated the feasibility and apparent safety of a specialized, home-care based protocol for the management of COVID-19 patients with moderate to high risk features. This included both ambulatory patients as well as post discharge COVID-19 patients who remained at moderate to high risk for decompensation and re-admission. Of our 182 patients, there were no unplanned hospital admissions or deaths. Two patients were referred for admission by our CROWN providers—one for respiratory distress and one for an INR of 13. Overall, seven patients went on their own to the ED, and were all treated and released without intervention—their symptoms largely felt to be due to anxiety.

Among the 101 not admitted patients, the majority (66%) were referred to us for acute or persistent dyspnea. The average age of referred patients was relatively young at 57 years old, majority female (74%), and had an average BMI of 30.2. Homecare evaluation and pulse oximeter was ordered by the CROWN provider in 35% of this group—though this number does not reflect a significant number of patients who had already been enrolled in homecare services by the referring primary care physician, as well as those who already owned a pulse oximeter. Oxygen was ordered for 12% of the patients, home-draw labs in 76% and intravenous fluid in 5% of patients. Prophylactic anticoagulation was ordered in 11% of patients based on elevated d dimer results, and 10% of patients were placed on prophylactic aspirin at physician discretion. None of the patients had a confirmed thromboembolic event during this time-period, though only 9% were evaluated for one. Seventy percent of the patients had a confirmed positive COVID-19 PCR, and overall 9% of patients were determined to not have COVID-19 based on both a negative PCR and IgG results. The average time from symptom onset to referral was 26 days, which was longer than expected given the danger zone of Day 7 to 10, however, this can be explained by the fact that by the time the CROWN program went into effect on April 27th, New York was in its second month of the pandemic and physicians did not have an option for patients prior to this program. In fact, after its second week in place, there was a trend to shorter time to referral, reflecting the more widespread familiarity with the program.

Among the 81 patients in the post-discharge group, the majority were referred due to persistent dyspnea (76%) and/or the need for home oxygen at discharge (53%). The average age in this group was 62 years old, and patients were evenly split female and male. Average BMI was 31, similar to the not admitted group. Home oxygen was ordered on two patients who had not received it upon discharge, home-care was ordered de-novo on 22 patients, labs were ordered on 59 and intravenous fluid for one patient. Seven of these patients had a confirmed thromboembolic event during the admission, and six were discharged on full dose anticoagulation. Fourteen patients were discharged on prophylactic dose anticoagulation. CTPA was ordered by a CROWN provider in eight patients, all of which showed bilateral ground glass opacities and one showed an acute pulmonary embolism in a patient who was not on prophylactic anticoagulation. Two patients were started on full-dose anticoagulation by the CROWN provider—the patient who was diagnosed with an acute pulmonary embolism, and the patient who was diagnosed with a pulmonary embolism during the admission but was erroneously discharged only with prophylactic dose. Seven additional patients were started on prophylactic dose anticoagulation by the CROWN provider based on d dimer results.

Our study has several limitations, most notably the length of time from symptom onset to CROWN referral, and the fact that New York was already past the peak of the COVID-19 infection curve by the time the protocol was implemented. However, given the ongoing surge of COVID-19 infections in other parts of the country and the significant possibility of resurgence in our area, having an already designed and implemented strategy for managing moderate to severe ambulatory patients remains very relevant now and for the future. Although our study was not randomized and had no comparison group, we were able to demonstrate the feasibility of enacting such a program. Finally, there was little known about COVID-19 as the program was implemented. As scientific studies are released on beneficial treatments, our suggested algorithm should adopt and change, including anticoagulation guidelines, antiviral therapies, and other treatments modalities.

Finally, it should be noted that although the Centers for Medicare and Medicaid Services expanded reimbursement for telehealth services in response to the COVID-19 pandemic, the majority of states, including New York, do not otherwise have a payment parity system for telemedicine.^4^ Continued progress on regulations and reimbursement for telehealth and homecare services is needed for protocols such as CROWN to grow.

## Conclusion

The CROWN program has demonstrated the feasibility and apparent safety of a specialized, homecare based protocol for the ambulatory management of moderate to high risk COVID-19 patients.
